# Improving skin cancer detection by Raman spectroscopy using convolutional neural networks and data augmentation

**DOI:** 10.3389/fonc.2024.1320220

**Published:** 2024-06-19

**Authors:** Jianhua Zhao, Harvey Lui, Sunil Kalia, Tim K. Lee, Haishan Zeng

**Affiliations:** ^1^ Photomedicine Institute, Department of Dermatology and Skin Science, University of British Columbia and Vancouver Coastal Health Research Institute, Vancouver, BC, Canada; ^2^ BC Cancer Research Institute, University of British Columbia, Vancouver, BC, Canada; ^3^ BC Children’s Hospital Research Institute, Vancouver, BC, Canada; ^4^ Centre for Clinical Epidemiology and Evaluation, Vancouver Coastal Health Research Institute, Vancouver, BC, Canada

**Keywords:** Skin cancer detection, Raman spectroscopy, convolutional neural networks (CNN), artificial intelligence (AI), optical diagnosis, data augmentation, machine learning

## Abstract

**Background:**

Our previous studies have demonstrated that Raman spectroscopy could be used for skin cancer detection with good sensitivity and specificity. The objective of this study is to determine if skin cancer detection can be further improved by combining deep neural networks and Raman spectroscopy.

**Patients and methods:**

Raman spectra of 731 skin lesions were included in this study, containing 340 cancerous and precancerous lesions (melanoma, basal cell carcinoma, squamous cell carcinoma and actinic keratosis) and 391 benign lesions (melanocytic nevus and seborrheic keratosis). One-dimensional convolutional neural networks (1D-CNN) were developed for Raman spectral classification. The stratified samples were divided randomly into training (70%), validation (10%) and test set (20%), and were repeated 56 times using parallel computing. Different data augmentation strategies were implemented for the training dataset, including added random noise, spectral shift, spectral combination and artificially synthesized Raman spectra using one-dimensional generative adversarial networks (1D-GAN). The area under the receiver operating characteristic curve (ROC AUC) was used as a measure of the diagnostic performance. Conventional machine learning approaches, including partial least squares for discriminant analysis (PLS-DA), principal component and linear discriminant analysis (PC-LDA), support vector machine (SVM), and logistic regression (LR) were evaluated for comparison with the same data splitting scheme as the 1D-CNN.

**Results:**

The ROC AUC of the test dataset based on the original training spectra were 0.886±0.022 (1D-CNN), 0.870±0.028 (PLS-DA), 0.875±0.033 (PC-LDA), 0.864±0.027 (SVM), and 0.525±0.045 (LR), which were improved to 0.909±0.021 (1D-CNN), 0.899±0.022 (PLS-DA), 0.895±0.022 (PC-LDA), 0.901±0.020 (SVM), and 0.897±0.021 (LR) respectively after augmentation of the training dataset (p<0.0001, Wilcoxon test). Paired analyses of 1D-CNN with conventional machine learning approaches showed that 1D-CNN had a 1–3% improvement (p<0.001, Wilcoxon test).

**Conclusions:**

Data augmentation not only improved the performance of both deep neural networks and conventional machine learning techniques by 2–4%, but also improved the performance of the models on spectra with higher noise or spectral shifting. Convolutional neural networks slightly outperformed conventional machine learning approaches for skin cancer detection by Raman spectroscopy.

## 1 Introduction

Skin cancers including basal cell carcinoma (BCC), squamous cell carcinoma (SCC) and malignant melanoma (MM) are the most common of all types of cancers with an estimate of over 5.4 million new skin cancer cases per year in the US (including 97,610 new melanoma cases) affecting more than 3.3 million patients (1). The incidence in Australia is even higher, with 2/3 of Australians developing skin cancer in their life time (2). Clinical diagnosis of skin cancer is typically based on visual inspection followed by an invasive biopsy of the suspicious lesion. It is invasive, time consuming and costly because the procedures of biopsy involve tissue processing and histology. Biopsies also generate a large number of false negatives and false positives. For example, in a large scale retrospective study of 4741 pigmented skin lesions, it was reported that for each confirmed melanoma, over 20 benign lesions were biopsied (3). Therefore, new techniques to aid skin cancer detection and reduce the misdiagnosis rate are being evaluated. A number of techniques have been proposed and different levels of performance have been demonstrated for skin cancer detection, such as Raman spectroscopy (4–7), dermoscopy (8–10), spectral imaging (11–13), confocal microscopy (14–16), electrical impedance spectroscopy (17, 18), multiphoton microscopy (19–22) and optical coherence tomography (OCT) (23).

Raman spectroscopy is an optical technique that measures the vibrational modes of biomolecules within the tissue. It is very sensitive to biochemical and biological changes associated with pathology. Raman spectroscopy has been investigated extensively for *in vitro* and *in vivo* skin cancer detection (4–7, 24–35). A number of excellent review articles on cancer detection by Raman spectroscopy have been published (36–40). Earlier work on skin cancer detection by Raman spectroscopy was limited either by *ex vivo* biopsied samples or by small number of *in vivo* cases due to long measurement times. For example, Gniadecka et al. (33) measured 223 punch biopsied skin samples by near infrared Fourier transform Raman spectroscopy, in which each spectrum was acquired over approximately 7 minutes. They found that the sensitivity and specificity for diagnosis of melanoma by neural network analysis were as high as 85% and 99%, respectively. Lieber et al. (27) measured 21 lesions and their adjacent normal skin *in vivo* with an integration time of 30 seconds, and reported 100% sensitivity and 91% specificity for discriminating skin lesions from normal skin. We have developed a rapid, real-time Raman spectrometer system for *in vivo* skin measurements that substantially reduced spectral acquisition times to less than a second (41, 42). In a recent study of 518 *in vivo* cases by our group, Lui et al. (4, 5) found that Raman spectroscopy could be used for skin cancer detection with an area under the receiver operating characteristic curve (ROC AUC) as high as 89.6% based on Raman spectrum alone. With feature selection (wavenumber selection) and by incorporating patient demographics into the algorithm, the diagnostic ROC AUC was further improved (6, 7). Very recently, Feng et al. quantified biophysical markers associated with different skin pathologies (24, 25). Bratchenko et al. (30–32) found that by combining Raman and autofluorescence spectra in the near-infrared region using a portable low-cost spectrometer, a reasonable diagnostic accuracy was achieved.

Recently Esteva et al. (43) reported that deep neural networks could improve the performance of skin cancer diagnosis based on color dermoscopic images. It stimulated further studies in artificial intelligence for biomedical image and spectral analysis (44–48). Currently, deep neural networks has been proposed for spectral analysis, such as spectral preprocessing (49–51), spectral classification (31, 52–56), and spectral data highlighting (57). Raman spectroscopy combining with deep neural networks have been reported for detection of breast cancer (biopsied samples, 8 subjects) (54, 58), colon cancer (*ex vivo* samples, 45 subjects) (53), prostate cancer (urine samples, 84 subjects) (55) and liver cancer (serum samples, 66 subjects) (59). All these studies were limited by the small number of cases, which might be over-trained for data-hungry deep neural networks that required a large amount of data to train. Bratchenko et al. (31) reported skin cancer detection using Raman spectroscopy and found that convolutional neural networks substantially improved the ROC AUC from 0.75 for PLS-DA to 0.96 for CNN based on the raw Raman spectra.

The objective of this study is to explore skin cancer detection by analyzing Raman spectra using deep neural networks. Based on clinical interest this study is focused on a dichotomous binary classification to determine whether a lesion is cancerous. We implemented different data augmentation strategies to increase the training dataset and compared the results of deep neural networks and conventional machine learning techniques with and without data augmentation. The paper is outlined as the following: section 2 described the patient dataset; different data augmentation strategies, in particular the details of one-dimensional generative adversarial networks (1D-GAN) for data augmentation; and the one-dimensional convolutional neural networks (1D-CNN) for spectral classification. Section 3 presented the performance of different data augmentation strategies and the results based on the original training datasets with and without data augmentation. Section 4 summarized the major findings and section 5 concluded the study.

## 2 Patient and method

### 2.1 Patient dataset

The dataset used in this study has been reported in a previous publication (7). In total, there were 731 valid lesions from 644 patients, including 326 males and 318 females with a median age of 62 years old (range: 18–94). Of the 731 lesions, 340 cases were cancerous or precancerous lesions (melanomas, basal cell carcinoma, squamous cell carcinoma and actinic keratosis), and 391 cases were benign lesions (atypical nevus, blue nevus, compound nevus, intradermal nevus, junctional nevus and seborrheic keratosis). All these lesions were clinically confirmed by the experienced dermatologists. All of the skin cancer lesions (100%), 29% of the precancer lesions and 34% of the benign lesions were also confirmed by histopathology. This study was approved by the Clinical Research Ethics Board of the University of British Columbia (Vancouver, BC, Canada; protocol C96–0499).

Raman spectra of all the lesions were measured *in vivo* using a custom-build real-time Raman spectrometer system (41, 42). The system contained a 785 nm diode laser, a hand-held Raman probe and a spectrograph equipped with liquid nitrogen cooled back-illumination deep depletion charge coupled device (CCD) detector. The laser was delivered to the Raman probe through a single multimode fiber with core diameter of 100 μm and formed a 3.5 mm diameter spot on the skin target. The Raman signal was collected by the Raman probe and delivered to the spectrograph through a fiber bundle, which consisted of 58 multimode optical fibers with core diameter of 100 μm. The distal end of the fiber bundle was packed into a circular area, and the proximal end connected to the spectrograph was aligned along a specially-designed parabolic line to correct the aberration of the spectrograph. Full-chip vertical hardware binning was achieved after image aberration correction, which improved the signal-to-noise ratio by 16 times (41, 42). The raw Raman signal was filtered by a 5-point box-car smoothing, and the fluorescence background was removed using fifth-order polynomial fitting of the Vancouver Raman Algorithm (60). Most of the lesions (96%) were acquired of a single spectrum; large and inhomogeneous lesions (4%) were acquired of multiple times from different locations within the lesion, and the averaged Raman spectrum was used for analysis. In this study, each individual lesion was considered as an experimental unit for analysis.

**Figure 1:**
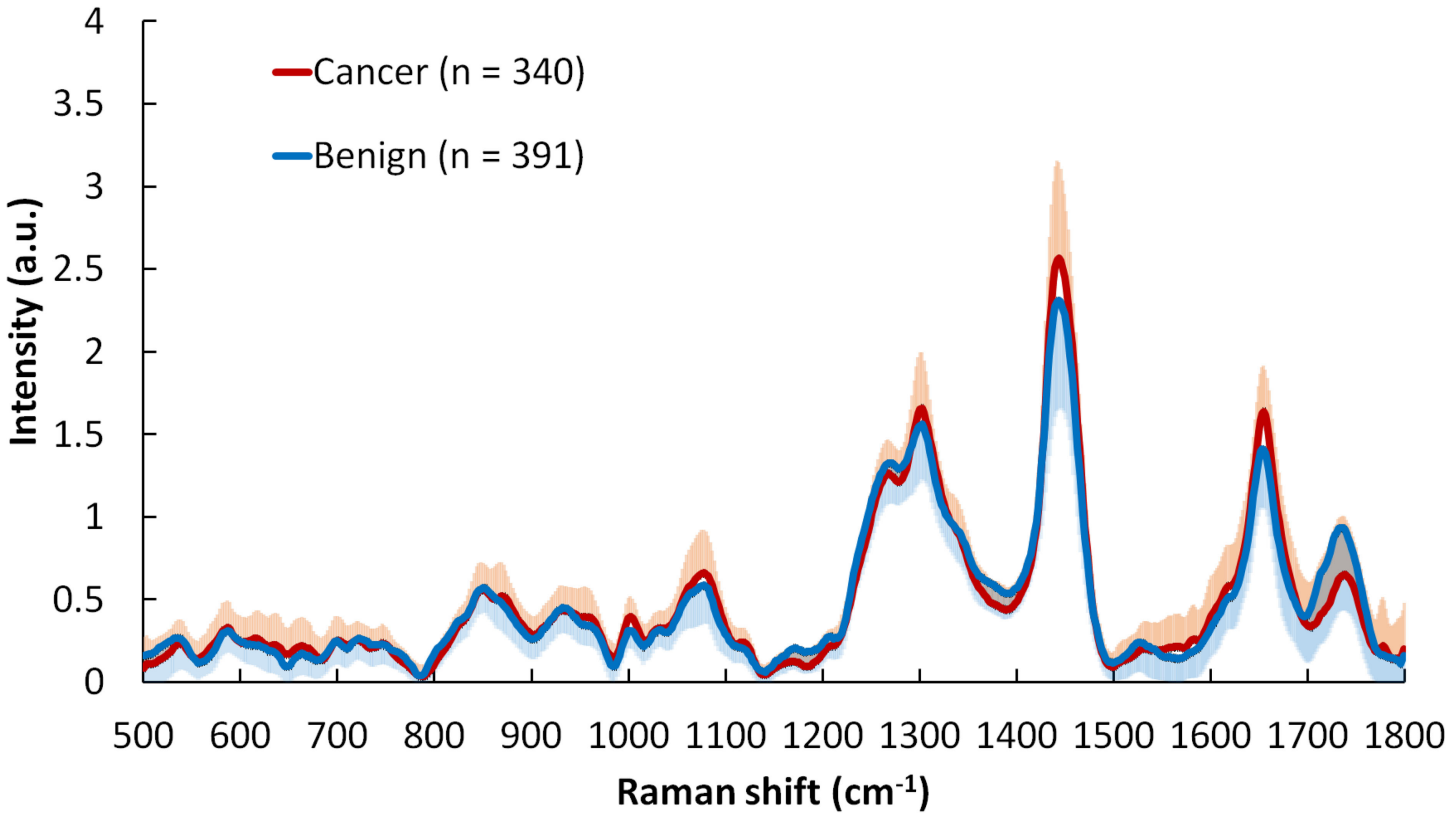
Averaged Raman spectra (and standard deviation) of malignant (n=340, including melanoma, basal cell carcinoma, squamous cell carcinoma and actinic keratosis) and benign skin lesions (n=391, including benign nevi and seborrheic keratosis). All the spectra were normalized to their respective areas under the curve between 500 and 1800 cm^−1^ before being averaged. For clarity, standard deviation is shown top half for cancer and bottom half for benign lesions.

The averaged Raman spectra and standard deviation of skin cancers and precancerous, and benign skin lesions were shown in **Figure 1**. All the spectra were normalized to their respective area under the curve between 500 and 1800 cm^−1^ before being averaged. Major Raman peaks were located around 855, 936, 1,002, 1,271, 1,302, 1,445, 1,655, and 1,745 cm^-1^. It was noted that all the skin lesions shared similar Raman peaks and bands with different intensities. These differences in intensities provided the diagnostic capability between skin cancers and benign skin lesions. It is difficult if not impossible to identify the peaks that can provide the best discrimination. Features extracted from machine learning techniques (such as principal components) and deep neural networks are used for classification (45), but generally difficult to interpret. A gradient-weighted class activation mapping (Grad-CAM) can be performed to highlight which regions contribute the most to the classification (61).

### 2.2 Data augmentation strategies

Deep neural networks require a large number of cases for training. Many data augmentation strategies were proposed for image analysis such as flipping, color space, translation, rotation, noise injection, image mixing, random cropping and generative adversarial networks (62–64). Different from images, the intensity of the Raman spectrum is highly dependent on the Raman shift (wavenumbers). Therefore, different data augmentation strategies are needed for one-dimensional spectral analysis. In previous studies, a number of data augmentation strategies for spectral analysis was proposed, including adding random noise (52, 53, 65), spectral shift (52, 53, 65), spectral superimposition (spectral linear combination) (52, 53, 65), offset (66), adding a slope (66), multiplication (66) and generative adversarial networks (GAN) (67). However, not all the data augmentation strategies were applicable for Raman spectroscopy. In this study, we proposed the following strategies for data augmentation of Raman spectra, including adding random noise, spectral shift, spectral linear combination, and artificially synthesized spectrum through generative adversarial networks. Note that data augmentation is conducted only for the training dataset.

#### 2.2.1 Data augmentation by addition of random noise

**Figure 2:**
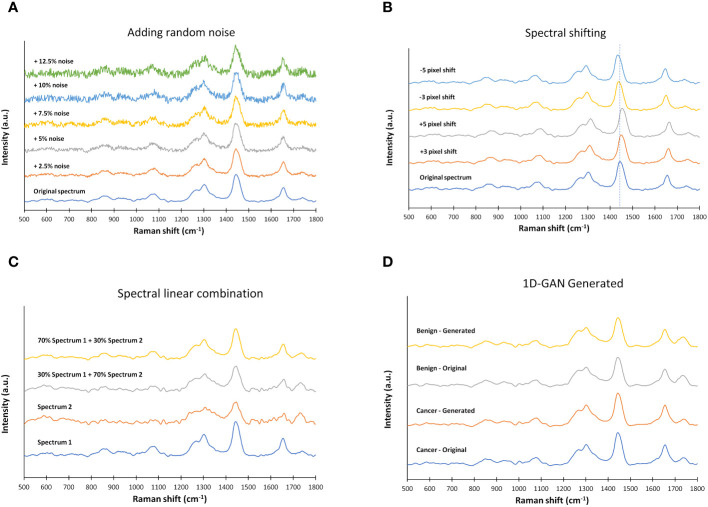
Examples of data augmentation for the training dataset of Raman spectra. **(A)** adding random noise of different noise levels, **(B)** spectral shifting, **(C)** spectral linear combination, and **(D)** data augmentation by one dimensional generative adversarial networks (1D-GAN) (averaged spectra are shown).


The training dataset can be augmented by adding random noise of different noise levels (**Figure 2A**), which can be written as

(1) $$S^{\prime }\left(v_{i}\right)=\;S\left(v_{i}\right)+\;N\left(v_{i}\right)$$

Where $S^{\prime }\left(v_{i}\right)$ is the augmented spectrum, $S\left(v_{i}\right)$ is the original spectrum, and $N\left(v_{i}\right)$ is the random noise. The random noise level is defined as the amplitude of the noise over the maximum peak intensity (*I_max_*) of the full training dataset. For example, a *k* percent of random noise is defined as *k* percent of the peak intensity, written as $N\left(v_{i}\right)=2\times \;\left(rand\left(\;\right)-0.5\right)\;\times \;k\;/\;100\times I_{max}$ , where *rand( )* is a random generator that produces uniformly distributed random numbers within the interval of (0,1).

#### 2.2.2 Data augmentation by spectral shift

The training dataset can be augmented by shifting the spectrum a few pixels (or wavenumbers) (**Figure 2B**), which can be written as


(2) $$S^{\prime }\left(v_{i}\right)=\;S\left(v_{i\;\pm \;m}\right)\;+N\left(v_{i}\right)$$

Where $S^{\prime }\left(v_{i}\right)$ the augmented spectrum at wavenumber $v_{i}$ , $S\left(v_{i\;\pm \;m}\right)$ is the original spectra at wavenumber $v_{i\;\pm \;m}$ , *m=1, 2, 3, …* and $N\left(v_{i}\right)$ is the random noise at wavenumber $v_{i}$ , generated by the same formula as shown in section 2.2.1.

#### 2.2.3 Data augmentation by spectral linear combination

The training dataset can also be augmented by linearly combining two or more sets of spectra. In this study, we implemented data augmentation by linearly combining two sets of spectra (**Figure 2C**), which can be written as


(3) $$S^{\prime }\left(v_{i}\right)=\;rS_{1}\left(v_{i}\right)+\;\left(1-r\right)S_{2}\left(v_{i}\right)+N\left(v_{i}\right)$$

Where $S^{\prime }\left(v_{i}\right)$ is the augmented spectrum at wavenumber $v_{i}$ , $S_{1}\left(v_{i}\right)$ and $S_{2}\left(v_{i}\right)$ are the two sets of randomly selected original spectra from the training dataset. *r* is a randomly generated number that is uniformly distributed between 0 and 1, representing the ratio of the two sets of the original spectra. Note that *S*_1_(*v_i_*) and $S_{2}\left(v_{i}\right)$ are randomly chosen from either the cancer group or the benign group. No attempt is tried to combine the spectra of one from the cancer and the other from the benign groups.

#### 2.2.4 Data augmentation by generative adversarial networks

**Figure 3:**
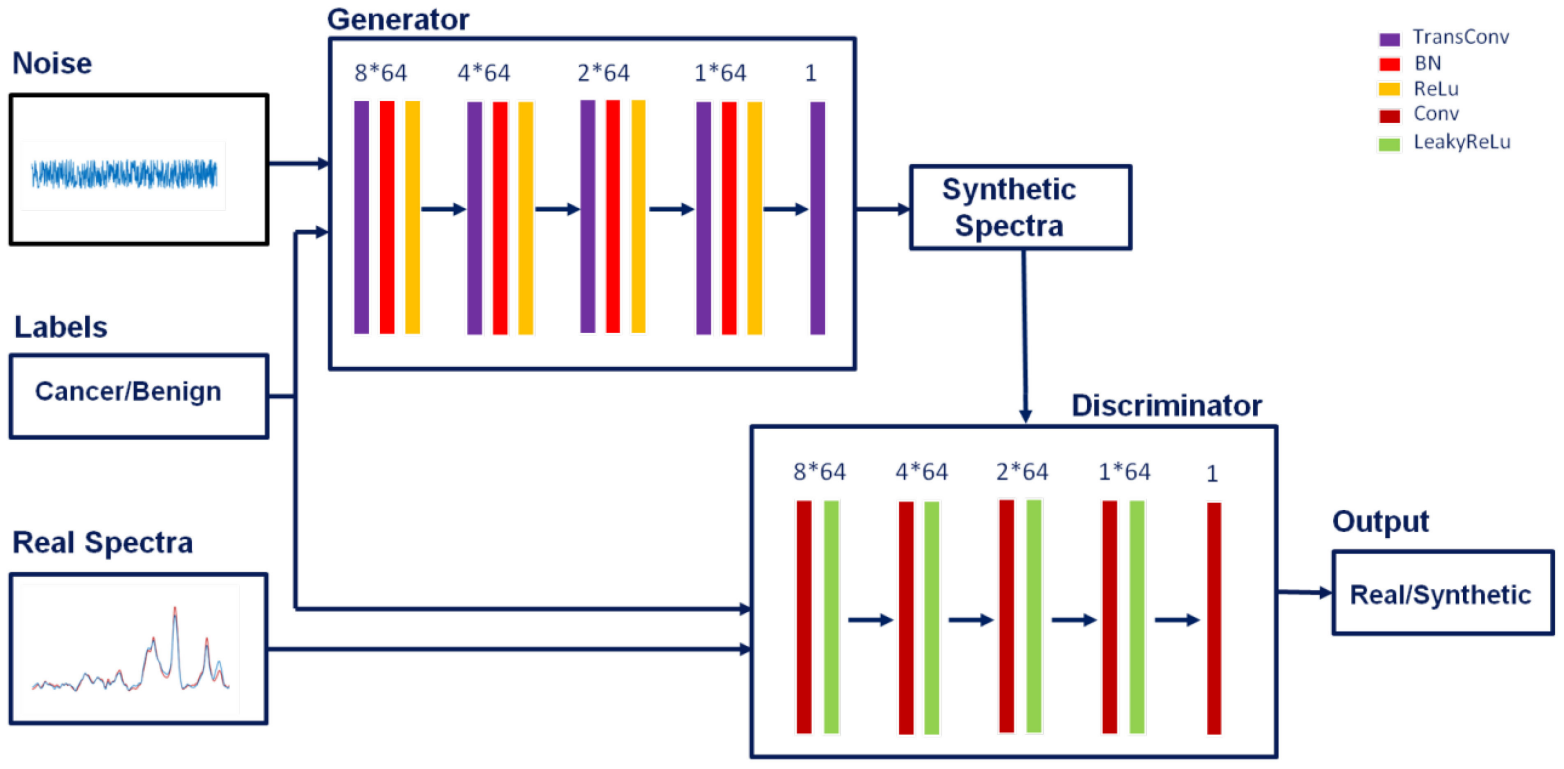
One-dimensional generative adversarial networks (1D-GAN) for data augmentation. 8*64, 4*64, 2*64, 1*64 and 1 are the number of kernels of each convolutional layer (and transposed convolutional layer for the generator).

Another way for data augmentation is using generative adversarial networks, which is far more complicated than the above simple data augmentation techniques. We designed a one-dimensional conditional generative adversarial network (1D-GAN) for Raman spectral generation as shown in **Figure 3**. It takes the general architecture of a conditional generative adversarial network, which contains two separate networks: a generator and a discriminator. The generator takes random noise and the label (cancer or benign) as input, and generates a synthetic spectrum. The discriminator takes the synthetic spectrum and label, and the real Raman spectra and labels as input, and tries to discriminate the synthetic spectrum from the real spectra. If the discriminator can separate the synthetic spectrum from the real spectra, it will provide a feedback to the generator to modify the parameters in such a way that the synthetic spectrum looks more like a real spectrum (decreasing the loss function). This process is iterative and eventually the generated spectrum looks so close to the real spectrum that the discriminator could not separate the synthetic spectrum from the real spectra. Once the 1D-GAN is trained, the generator can be used to generate arbitrary number of synthetic Raman spectra based on random input (67). The discriminator could sometimes be used directly for image and spectral discrimination purpose as well (68, 69).

The architectures of the generator and discriminator are shown in **Figure 3** and the parameters are listed in **Table 1**. The generator contains 5 transposed convolutional layers (TransConvolution). Each of the first 4 transposed convolutional layers is followed by a batch normalization layer and a regularization layer (ReLu). The output size of each layer is governed by *o* = (*i-*1) *s* – 2*p* + *k*, where *o* is the output size, *i* is the input size, *s* is the stride, *p* is the padding size, and *k* is the kernel size (70). Assuming the input size is 4, the output size of the generator is 619, which is the length of the spectrum in this study within the range of 500 – 1800 cm^-1^.

**Table 1:** Parameters for the generator and discriminator of the one-dimensional generative adversarial networks (1D-GAN) for data augmentation.

Network	Input size	Layer number	Kernel size	Number of kernels	Stride	Cropping	Output size
**Generator**	4	1	[3,1]	8 * 64	[1,1]	[0,0]	6
	6	2	[7,1]	4 * 64	[4,1]	[1,0]	25
	25	3	[7,1]	2 * 64	[3,1]	[1,0]	77
	77	4	[7,1]	1 * 64	[4,1]	[1,0]	309
	309	5	[5,1]	1	[2,1]	[1,0]	619
**Discriminator**	619	1	[5,1]	8 * 64	[2,1]	[1,0]	309
	309	2	[7,1]	4 * 64	[4,1]	[1,0]	77
	77	3	[7,1]	2 * 64	[3,1]	[1,0]	25
	25	4	[7,1]	1 * 64	[4,1]	[1,0]	6
	6	5	[6,1]	1	[1,1]	[0,0]	1

The input size of the generator is designed as 4 and the output size of the generator is 619.

The discriminator also contains 5 convolutional layers. Each of the first 4 convolutional layers was followed by a regularization layer (LeakyReLu). The output size of each layer is governed by *o* = (*i* + 2*p* – *k*)/*s* + 1, where *o* is the output size, *i* is the input size, *s* is the stride, *p* is the padding size, and *k* is the kernel size (70). The output size of the discriminator is 1, indicating the input spectrum is either real or synthesized after the discriminator.

The mini batch size was 256. The initial learning rate was 0.0002. The gradient decay factor and the squared gradient decay factor was 0.5 and 0.999 respectively. The total number of epochs was 25,000. With such parameters for the above 1D-GAN architecture, it took about 4.5 hours to complete the training using a mainframe GPU (Advanced Research Computing, University of British Columbia, Sockeye high-performance computing platform). After the 1D-GAN was trained, 5,000 spectra were generated for skin cancers and 5,000 spectra were generated for benign lesions. The average of the 1D-GAN generated spectra and the average of real spectra were shown in **Figure 2D**.

### 2.3 One dimensional convolutional neural networks for spectral classification

**Figure 4:**
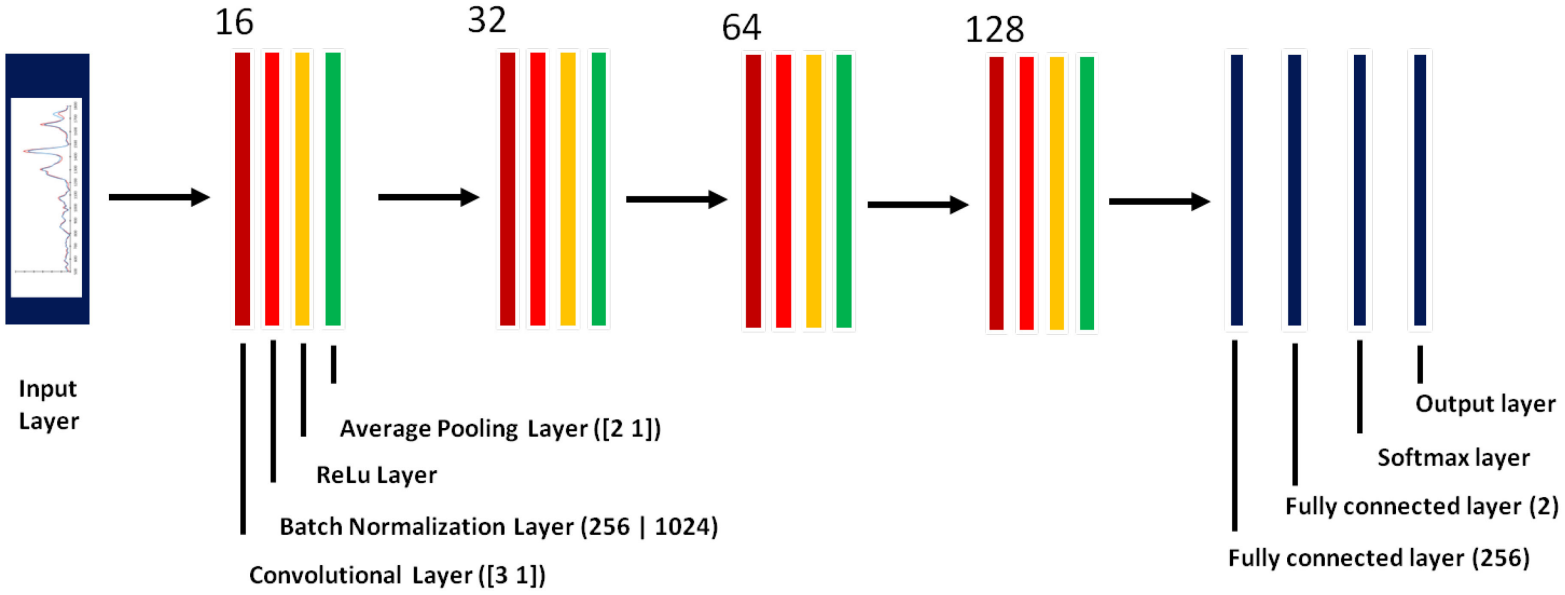
Architecture and parameters of one-dimensional convolutional neural networks (1D-CNN) for Raman spectral classification. The number of kernels for each convolutional layer was 16, 32, 64 and 128 with kernel size = [3,1] for all the convolutional layers. The mini-batch size was 256 for the training dataset without augmentation and 1024 for the training dataset with augmentation. The pooling size was [2,1] with stride [2,1] for each average pooling layer. The size for the first fully connected layer was 256 and for the second fully connected layer was 2.

We developed and tested a number of 1D-CNN architectures for Raman spectral classification, including different number of convolutional layers (1–5); number of kernels (16, 32, 64, 128) for each convolutional layer; kernel sizes (3, 5, 7, 9); mini-batch sizes (16, 32, 64, 128, 256); pooling methods (max pooling and average pooling); and sizes of the fully connected layers (128, 256, 512) (**Supplementary Tables S1–S4**). The final architecture of the designed 1D-CNN for Raman spectral classification that provided the best performance contained an input layer, 4 convolutional layers, 2 fully connected layers, a softmax layer and an output layer (**Figure 4**). Each of the four convolutional layers was followed by a batch normalization layer, a regularization layer (ReLu) and an average pooling layer. In total, the 1D-CNN had 21 layers. Note that each layer represents a specific data manipulation. The four convolutional layers had the same kernel (filter) size, padding and stride (kernel size = [3, 1], padding = ‘same’, and stride = 1), but with different number of kernels (16, 32, 64, and 128 respectively). Zero padding was added to each convolutional layer (padding =‘same’) so that the output of each convolutional layer had the same size as the input. The size of batch normalization layer was 256 for the original training dataset and 1024 for the augmented training dataset. The four average pooling layers had the same parameters (size = [2,1], stride= [2,1]) so that after each pooling layer the size was reduced by half. The training process was optimized by adaptive moment estimation (adam) (71). The initial learning rate was 0.001, which was dropped by a factor of 0.9 for every 2 period. The training process was monitored through the accuracy and the loss function (cross entropy) of the training and validation datasets.

**Figure 5:**
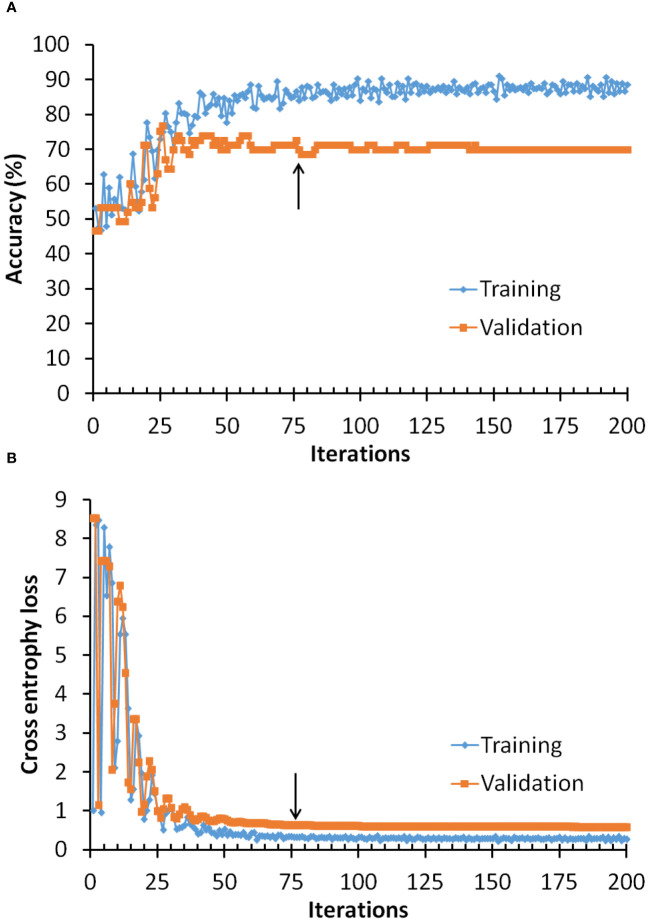
Example of the training process of the 1D-CNN for Raman spectral classification. Arrows showed the performance of the validation process no longer improving over at least 50 iterations, a possible stopping stage to prevent over-training. **(A)** Accuracy of the training and validation process. **(B)** Cross entropy loss of the training and validation process.

An example of the training process, including the accuracy and the loss function of the training and validation processes are shown in **Figure 5**. Here the original training dataset was used, which contained 512 cases. The mini-batch size was 256, and the maximum number of epochs was 100. Therefore, there were 2 iterations per epoch and a maximum of 200 iterations per training process in this example. The accuracy for the training dataset was defined as the ratio of the correctly classified cases to the total cases of the training dataset in the mini-batch (n=256). The accuracy for the validation dataset was defined as the ratio of the correctly classified cases to the total cases of the full validation dataset (n=73). It was noted that the accuracy of the model was improving at the beginning after the network training process started. However, the model may be over-trained if the training process could not be terminated at appropriate training stage. To prevent over-training, we implemented a strategy to stop the training process if the accuracy of the validation dataset was not improving for specific iterations. Usually the number of iterations for terminating the training process could be set between 10 and 50. If the number was too small, the trained model might be premature; while if the number was too large, the model might be over-trained. **Figure 5A** shows that the model was terminated at 76 iterations (as shown by the arrow) because the accuracy of validation dataset was not improving for 50 iterations. The parameters of last training process were used as the parameters for the final model. **Figure 5B** shows the cross entropy loss of the training and validation process. Similarly to the accuracy, the cross entropy loss was calculated on mini-batch for the training process and on the full validation dataset for the validation process. It could be seen that the loss was initially decreased and then was leveled off if the model kept training. The arrow indicated a possible stopping stage based on the strategy for the training process to prevent over-training. In our experiment, we terminated the training process if the accuracy for the validation dataset was not improving for 50 iterations.

### 2.4 Conventional machine learning approaches

Conventional machine learning approaches are sometimes called chemometrics or multivariate statistical analyses (48, 72). For comparison purpose, conventional machine learning techniques, including partial least squares for discriminant analysis (PLS-DA), principal component and linear discriminant analysis (PC-LDA), support vector machine (SVM), and logistic regression (LR) were implemented.

### 2.5 Dataset split and data augmentation

**Figure 6:**
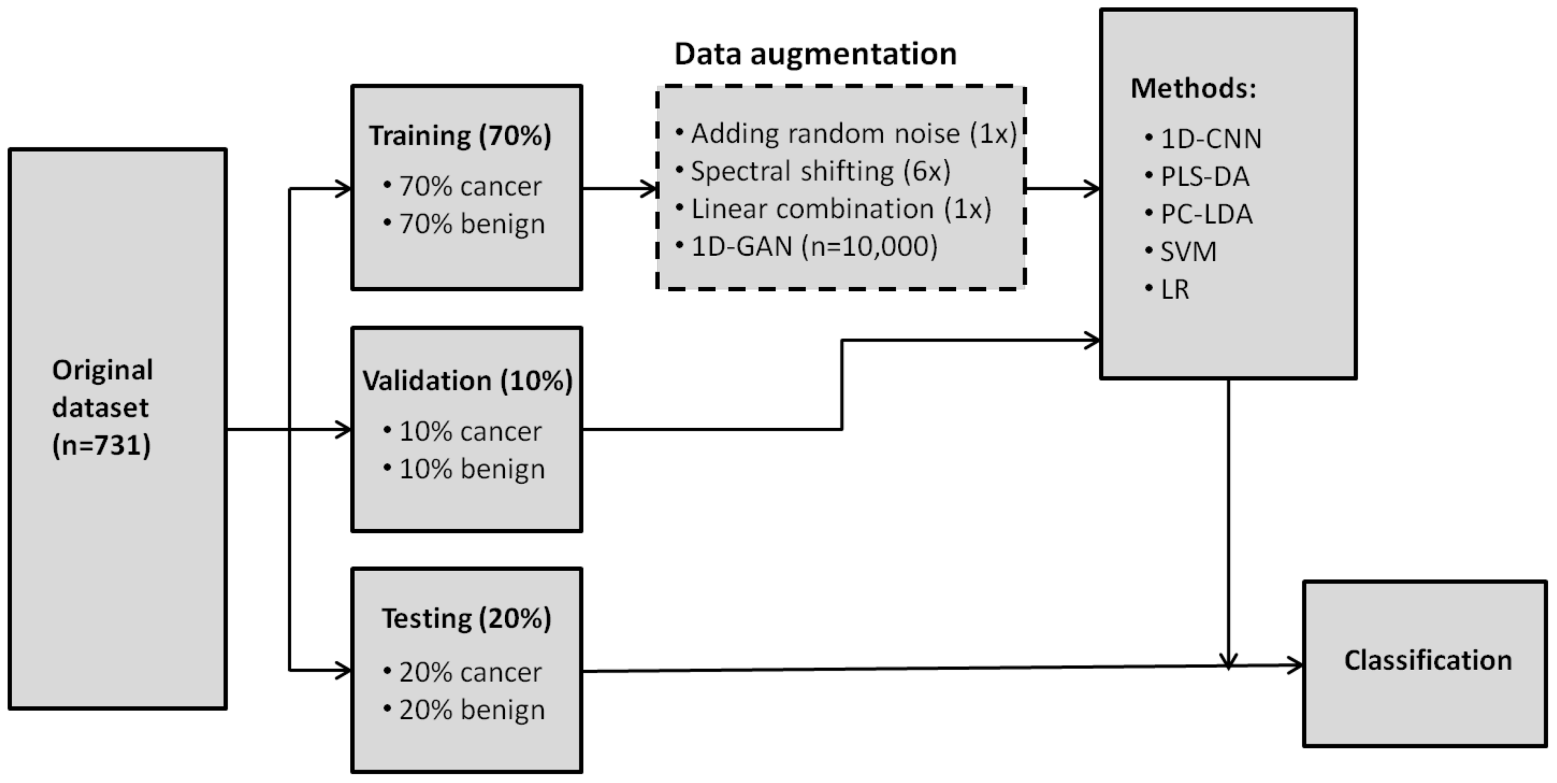
Random split of original dataset into training (70%), validation (10%) and testing (20%) datasets with and without data agumentation (dashed frame). Note that for analyses with augmentation, it was implemented only to the training dataset, not the validation and testing datasets. 1D-CNN, one-dimensional convolutional neural networks; PLS-DA, partial least squares for discriminant analysis; PC-LDA, pricipal component and linear discriminant analysis; SVM, support vector machine; LR, logistic regression.

The dataset split and data augmentation procedures were illustratively shown in **Figure 6**. For analyses without data augmentation, the stratified original dataset (n=731) was randomly divided into training (70%, i.e. 70% of cancerous and 70% of benign cases), validation (10%) and test set (20%). For analyses with data augmentation, only the training set was augmented. Data augmentation of the training set was implemented after random split of the original dataset. Therefore, the cases in the training, validation and test datasets were the same as the analyses without data augmentation. The same data split scheme and augmentation were used for the 1D-CNN and all the conventional machine learning analyses. All the above models including 1D-CNN, PLS-DA, PC-LDA, SVM and LR were implemented using parallel computing on UBC ARC (Advanced Research Computing, University of British Columbia) Sockeye high-performance computing platform. The random split was repeated in parallel 56 times and the mean was reported (parallel computing requires multiple of 8). All the programs were implemented using Matlab (Version 2021a, Mathworks, Natick, MA, USA).

### 2.6 Extended test dataset

In order to evaluate the models for situations that are slightly out of the original scope, such as lower spectral quality, we introduced random noise and spectral shift to the test dataset, hereafter referred as extended test dataset, in addition to the original test dataset. Similar to sections 2.2.1 and 2.2.2, up to 12.5% of random noise and 6-pixel shift were applied to the original test dataset to generate the extended test dataset.

### 2.7 Statistical analysis

Paired analysis (Wilcoxon test) of the test set between 1D-CNN and PLS-DA, PC-LDA, SVM and LR, and the test of the above models between using original and augmented training set were performed (GraphPad, Boston, MA, USA). A p-value of less than 0.05 (p<0.05) was regarded as statistically significant.

## 3 Results

### 3.1 Evaluation of augmentation parameters

We first evaluated the optimal parameters for data augmentation, such as the level of random noise, the range of the spectral shift, the number of linearly combined spectra, and the number of synthesized spectra generated by the generative adversarial networks.

#### 3.1.1 Level of random noise

**Figure 7:**
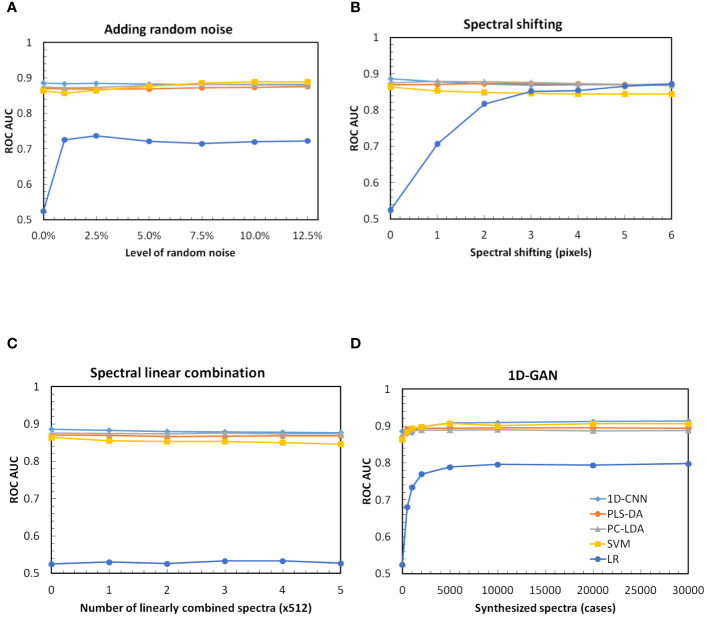
ROC AUC of the test dataset of 56 random repetitions based on the original training dataset and different augmentation parameters, **(A)** adding random noise, **(B)** spectral shifting, **(C)** spectral linear combination, and **(D)** synthesized spectra by 1D-GAN. 1D-CNN, one-dimensional convolutional neural networks; PLS-DA, partial least squares for discriminant analysis; PC-LDA, principal component and linear discriminant analysis; SVM, support vector machine; LR, logistic regression.

The hypothesis for data augmentation by adding random noise is that the augmented spectra are measured by systems of different signal to noise ratios. We evaluated data augmentation by adding different levels of random noise from 1% to 12.5% following Equation 1 (**Figure 7A**). As expected, the performances of 1D-CNN, PLS-DA and PC-LDA were immune to noise levels. Surprisingly, the performances of SVM and LR were all improved. The performance of SVM after augmentation with high level of noise (i.e. >7.5%) was even better than any other techniques. Because the number of cases was less than the number of variables for the original training set, data augmentation by adding random noise improved the performance of LR. However, data augmentation by solely adding random noise could not solve the number of case versus variable issues (full rank issues). Its performance was still the lowest compared with other techniques.

#### 3.1.2 Range of spectral shifting

The hypothesis for data augmentation by spectral shifting is that the augmented spectra are measured from different systems of variable qualities. We evaluated data augmentation by spectral shifting of 1 to 6 pixels following Equation 2 without addition of random noise (**Figure 7B**). It was found that the performance of 1D-CNN, PLS-DA and PC-LDA were independent of spectral shifting, while the performance of SVM was decreased monotonically. Surprisingly, it was found that data augmentation by spectral shifting was particularly useful for LR. Its performance was equivalent to 1D-CNN, PLS-DA and PC-LDA, and even better than SVM after data augmentation with large spectral shifting.

#### 3.1.3 Number of cases by spectral linear combination

The hypothesis for data augmentation by spectral linear combination is that the augmented spectrum is measured from a lesion that mimics two measured lesions. We evaluated data augmentation by spectral linear combination in multiples of the training dataset following Equation 3 (**Figure 7C**). It was found that the performance of spectral linear combination was the worst compared to data augmentation by adding random noise or spectral shifting, indicating that it was very unlikely that a lesion would have the properties of two measured lesions. Data augmentation by solely spectral linear combination did not improve the performance of 1D-CNN, PLS-DA, PC-LDA, SVM or LR.

#### 3.1.4 Number of synthesized spectra by 1D-GAN

The hypothesis of data augmentation by 1D-GAN is that the properties of a lesion that is not in the original dataset can be synthesized. The beauty of 1D-GAN is that once it is trained, it can be used to generate any number of synthesized spectra. To determine the optimal number of synthesized spectra by 1D-GAN, models with the original spectra and synthesized spectra of n=500, 1,000, 2,000, 5,000, 10,000, 20,000 and 30,000 were evaluated (**Figure 7D**). It was found that the performance of 1D-CNN, PLS-DA, PC-LDA, SVM and LR were all improved with augmentation by 1D-GAN generated spectra. However, PLS-DA, PC-LDA and SVM were not dependent on the number of synthesized cases. 1D-CNN was slightly improved with the number of synthesized spectra until it reached plateau at around n=10,000, while for LR no matter how many cases were synthesized, it was not sufficient.

#### 3.1.5 Optimal parameters for data augmentation

Based on the above evaluation, the following parameters were selected for data augmentation of the training set in this study: random noise level of 5% (n=512), spectral shift of 1–3 pixels (n=512x6), spectral linear combination (n=512) and 1D-GAN synthesized spectra (n=10,000). To prevent collinearity, a 5% random noise was applied to the spectrally shifted and linearly combined spectra.

### 3.2 Diagnosis based on original training dataset without data augmentation

**Figure 8:**
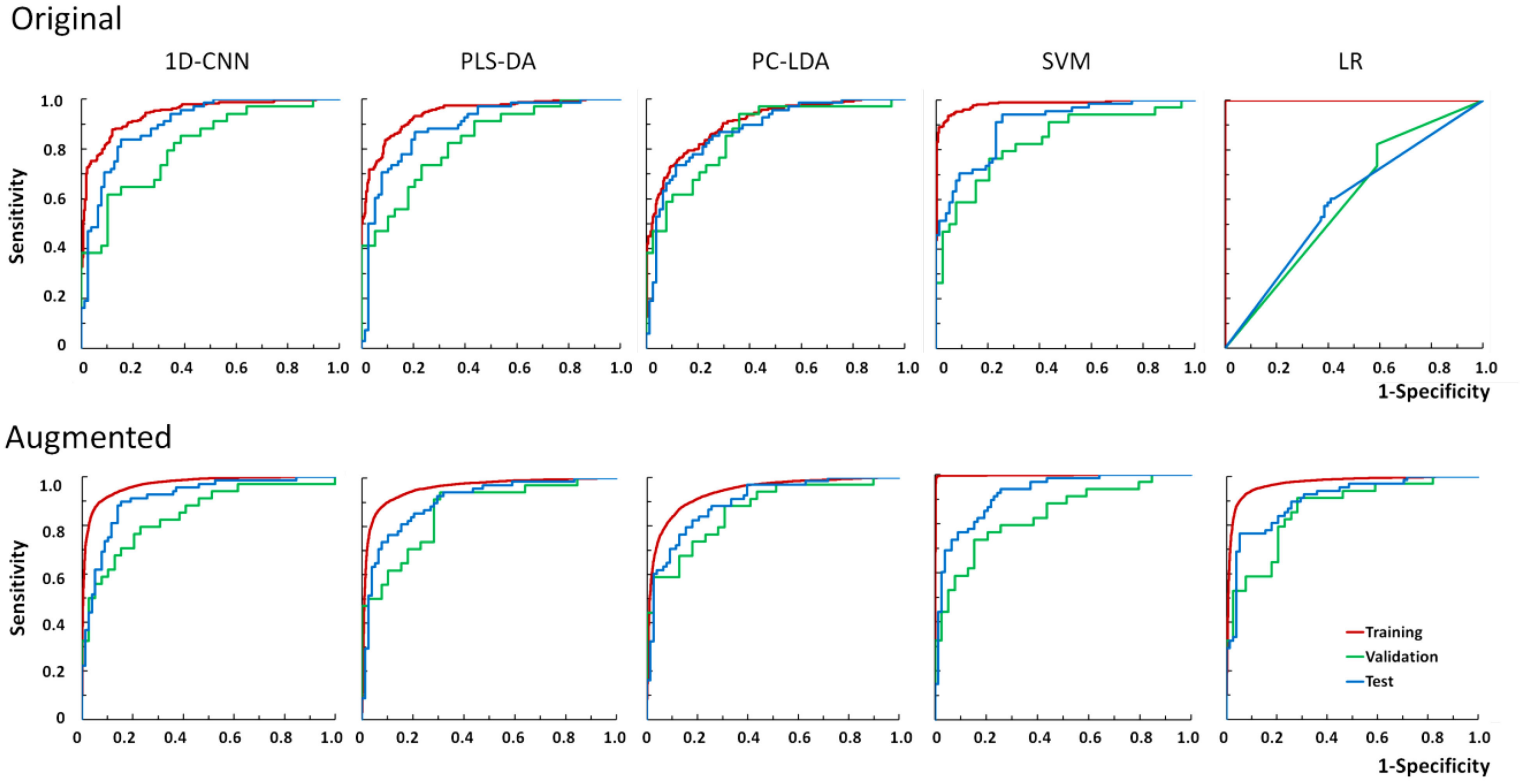
Example of the ROC curves of the training, validation (n=73) and test (n=146) datasets. Top row is based on the original training spectra (n=512), and bottom row is based on the augmented spectra (n=14,608). 1D-CNN, one-dimensional convolutional neural networks; PLS-DA, partial least squares for discriminant analysis; PC-LDA, principal component and linear discriminant analysis; SVM, support vector machine; LR, logistic regression.

The ROC curves for the training, validation and test dataset based on 1D-CNN, PLS-DA, PC-LDA, SVM and LR for one of the 56 random splits are shown in **Figure 8** (top row). It could be seen that the performance of the training set were always better than the validation and the test sets, indicating that the models were slightly over-trained, particularly for SVM. LR failed based on the original training set in this example, because the number of variables (619) was more than the number of cases (512).

**Figure 9:**
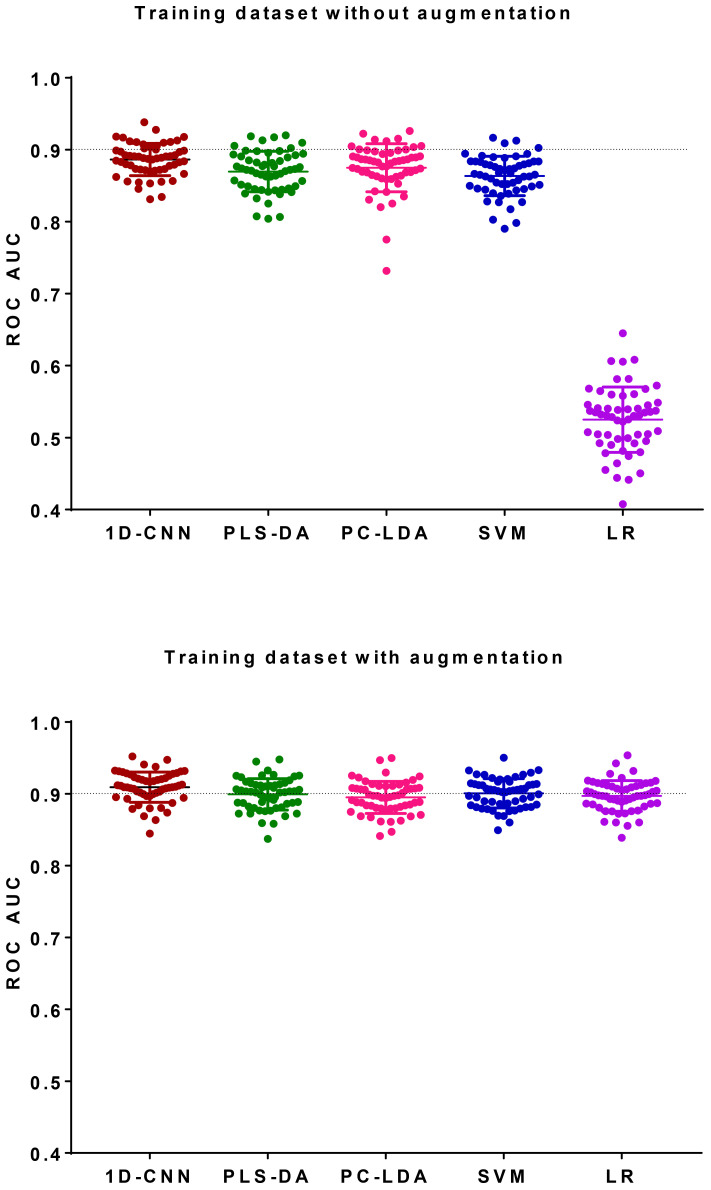
ROC AUC of the test dataset of 56 random repetitions based on the original training dataset without augmentation and the original training dataset with augmentation. Bar shows the mean and standard deviation.

The ROC AUCs of the 56 calculations for the original test datasets based on the original training datasets without augmentation were shown in **Figure 9**. The training set had higher ROC AUCs than the validation and test datasets on average (**Table 2**). The averaged ROC AUCs of the original *test dataset* based on the original training spectra without augmentation were 0.886±0.022 (1D-CNN), 0.870±0.028 (PLS-DA), 0.875±0.033 (PC-LDA), 0.864±0.027 (SVM), and 0.525±0.045 (LR), respectively. Paired analyses showed that 1D-CNN outperformed conventional machine learning approaches by 1–3% including PLS-DA, PC-LDA and SVM based on the original spectra (Wilcoxon, p<0.001). 1D-CNN also had the smallest standard deviation of the ROC AUCs.

**Table 2:** ROC AUCs (mean ± standard deviation) of the training, validation (n=73) and test dataset (n=146) of 56 random split schemes based on models of different training datasets.

Training Dataset	Dataset	1D-CNN	PLS-DA	PC-LDA	SVM	LR
**Original without augmentation**	Training	0.941±0.005	0.911±0.030	0.910±0.025	0.980±0.006	0.999±0.001
	Validation	0.882±0.041	0.889±0.039	0.900±0.036	0.853±0.043	0.541±0.058
	Test	**0.886±0.022**	**0.870±0.028**	**0.875±0.033**	**0.864±0.027**	**0.525±0.045**
**Original with augmentation**	Training	0.989±0.005	0.961±0.002	0.949±0.011	0.999±0.000	0.980±0.002
	Validation	0.903±0.042	0.895±0.036	0.909±0.035	0.893±0.039	0.889±0.037
	Test	**0.909±0.021**	**0.899±0.022**	**0.895±0.022**	**0.901±0.020**	**0.897±0.021**
**Augmented spectra without original**	Training	0.989±0.005	0.963±0.002	0.949±0.015	0.999±0.000	0.981±0.002
	Validation	0.902±0.040	0.895±0.037	0.909±0.035	0.897±0.039	0.889±0.037
	Test	**0.908±0.021**	**0.899±0.022**	**0.893±0.023**	**0.906±0.021**	**0.897±0.022**
**Augmented spectra by 1D-GAN only**	Training	1.000±0.001	0.993±0.000	0.980±0.021	1.000±0.000	1.000±0.000
	Validation	0.902±0.038	0.890±0.040	0.912±0.033	0.903±0.034	0.810±0.047
	Test	**0.907±0.021**	**0.893±0.023**	**0.886±0.025**	**0.905±0.021**	**0.813±0.033**
**Original and augmentation without 1D-GAN**	Training	0.946±0.010	0.931±0.007	0.905±0.016	0.995±0.001	0.955±0.006
	Validation	0.876±0.043	0.881±0.039	0.895±0.037	0.866±0.043	0.870±0.041
	Test	**0.879±0.022**	**0.884±0.023**	**0.880±0.026**	**0.877±0.024**	**0.875±0.022**
**Augmentation without 1D-GAN**	Training	0.947±0.011	0.931±0.007	0.905±0.017	0.992±0.002	0.956±0.006
	Validation	0.873±0.043	0.881±0.039	0.894±0.038	0.872±0.043	0.871±0.041
	Test	**0.876±0.022**	**0.884±0.023**	**0.880±0.026**	**0.880±0.023**	**0.876±0.022**

Original without augmentation: original training spectra without augmentation (n=512); Original with augmentation: original training spectra with augmentation including adding random noise, spectral shifting, spectral linear combination and 1D-GAN (n=14,608); Augmented spectra without original: augmentation including adding random noise, spectral shifting, spectral linear combination and 1D-GAN but without original spectra (n=14,096); Augmented spectra by 1D-GAN only: augmented training spectra by 1D-GAN only (n=10,000); Original and augmentation without 1D-GAN (n=4608): Original training spectra with augmentation including adding random noise, spectral shifting, spectral linear combination but without 1D-GAN; Augmentation without 1D-GAN (n=4096): augmented spectra including adding random noise, spectral shifting, spectral linear combination but without 1D-GAN. Boldface highlights the results of the test dataset.

### 3.3 Diagnosis based on original training dataset with augmentation

There were 14,608 cases in the training dataset after augmentation, consisting of the original spectra (n=512) plus spectra that were augmented by adding random noise (5% noise, n=512), spectral shifting (1–3 pixel shifting, n = 512 x 6), combining spectra linearly (n=512), and 1D-GAN (n=10,000). The ROC curves based on same random split of the original dataset after augmentation for 1D-CNN, PLS-DA, PC-LDA, SVM and LR are shown in **Figure 8** (bottom row). It showed that all the models for the training dataset were also over-trained, particularly for SVM. Surprisingly, it was found that after data augmentation, LR performed very well since the number of cases was now larger than the number of variables. As expected data augmentation also resulted in smoother training ROCs.

The ROC AUCs of the 56 calculations for the original test datasets based on the original training datasets with augmentation are shown in **Figure 9**. The ROC AUCs of the training dataset were much higher than the validation and test datasets (**Table 2**).The ROC AUCs of the *original test dataset* based on the original training dataset with augmentation were 0.909±0.021 (1D-CNN), 0.899±0.022 (PLS-DA), 0.895±0.022 (PC-LDA), 0.901±0.020 (SVM), and 0.897±0.021 (LR). It showed that after data augmentation, 1D-CNN slightly out-performed all the conventional machine learning techniques by 1–2%, including PLS-DA, PC-LDA, SVM and LR (p<0.001, Wilcoxon test).

Because only the training datasets were augmented, the test datasets were the same for 1D-CNN and conventional machine learning approaches. Paired analyses demonstrated that models based on the original training datasets with augmentation (n=14,608) significantly improved the diagnostic ROC AUCs of the original test datasets by 2–4% compared with models based on the original training datasets without augmentation (n=512) for both 1D-CNN and conventional machine learning methods (PLS-DA, PC-LDA, and SVM) (p<0.0001, Wilcoxon test). Augmentation was particularly useful for LR when the number of cases was smaller than the number of variables, which was improved by 71%.

### 3.4 Diagnosis based on different augmentation strategies

We also calculated the performance of models based on different augmentation strategies to the training datasets. The split scheme of the original spectra was the same as section 3.2 and section 3.3. Validation and test datasets were not augmented. All the models were repeated 56 times.

#### 3.4.1 Augmented spectra without original

When 1D-CNN and the conventional machine learning approaches were trained on the augmented spectra without the original training datasets, here data augmentation included adding random noise (n=512), spectral shifting (n = 512 x 6), combining spectra linearly (n=512), and 1D-GAN (n=10,000), the ROC AUCs of the *original test datasets* were found to be 0.908±0.021 (1D-CNN), 0.899±0.022 (PLS-DA), 0.893±0.023 (PC-LDA), 0.906±0.021 (SVM), and 0.897±0.022 (LR) (**Table 2**), almost identical to the results based on the original training datasets with augmentation (section 3.3), indicating that the contribution of the original training datasets may be negligible after data augmentation.

#### 3.4.2 Augmented spectra by 1D-GAN only

When 1D-CNN and the conventional machine learning approaches were trained on the augmented spectra synthesized by 1D-GAN only (n=10,000), the ROC AUCs of the *original test datasets* were found to be 0.907±0.021 (1D-CNN), 0.893±0.023 (PLS-DA), 0.886±0.025 (PC-LDA), 0.905±0.021 (SVM), and 0.813±0.033 (LR) (**Table 2**). The results were inferior to the models based on the original training datasets with augmentation (section 3.3) and augmented spectra without original (section 3.4.1), but still better than the models based on the original training datasets without augmentation (section 3.2). However, the performance based on augmented spectra by 1D-GAN only was not sufficient for LR, indicating that other augmentation strategies were still needed.

#### 3.4.3 Original and augmentation without 1D-GAN

When the models were trained on the original training datasets with augmentation by adding random noise, spectral shifting and linear combination without 1D-GAN (n=4,608), the ROC AUCs of the *original test datasets* were found to be 0.879±0.022 (1D-CNN), 0.884±0.023 (PLS-DA), 0.880±0.026 (PC-LDA), 0.877±0.024 (SVM), and 0.875±0.022 (LR) (**Table 2**). The results indicated that data augmentation by adding random noise, spectral shifting and linear combination without 1D-GAN worked well for conventional machine learning techniques, particularly LR, but not 1D-CNN.

#### 3.4.4 Augmentation without 1D-GAN

When the models were trained on the augmented datasets (by adding random noise, spectral shifting and linear combination without 1D-GAN) without the original training datasets (n=4,096), the ROC AUCs of the *original test datasets* were found to be 0.876±0.022 (1D-CNN), 0.884±0.023 (PLS-DA), 0.880±0.026 (PC-LDA), 0.880±0.023 (SVM), and 0.876±0.022 (LR) (**Table 2**). These results were quite similar to the models trained on the original and augmentation without 1D-GAN (section 3.4.3), indicating that data augmentation by adding random noise, spectral shifting and linear combination without 1D-GAN covers the original training datasets. It is interesting to note that data augmentation without 1D-GAN worked better for LR than augmented spectra by 1D-GAN only (p<0.0001, Wilcoxon); while it was the opposite for 1D-CNN, PLS-DA, PC-LDA and SVM where augmented spectra by 1D-GAN only worked better (p<0.0001, Wilcoxon) (section 3.4.2).

### 3.5 Diagnosis on extended test dataset

#### 3.5.1 Models based on the original training dataset without augmentation

**Figure 10:**
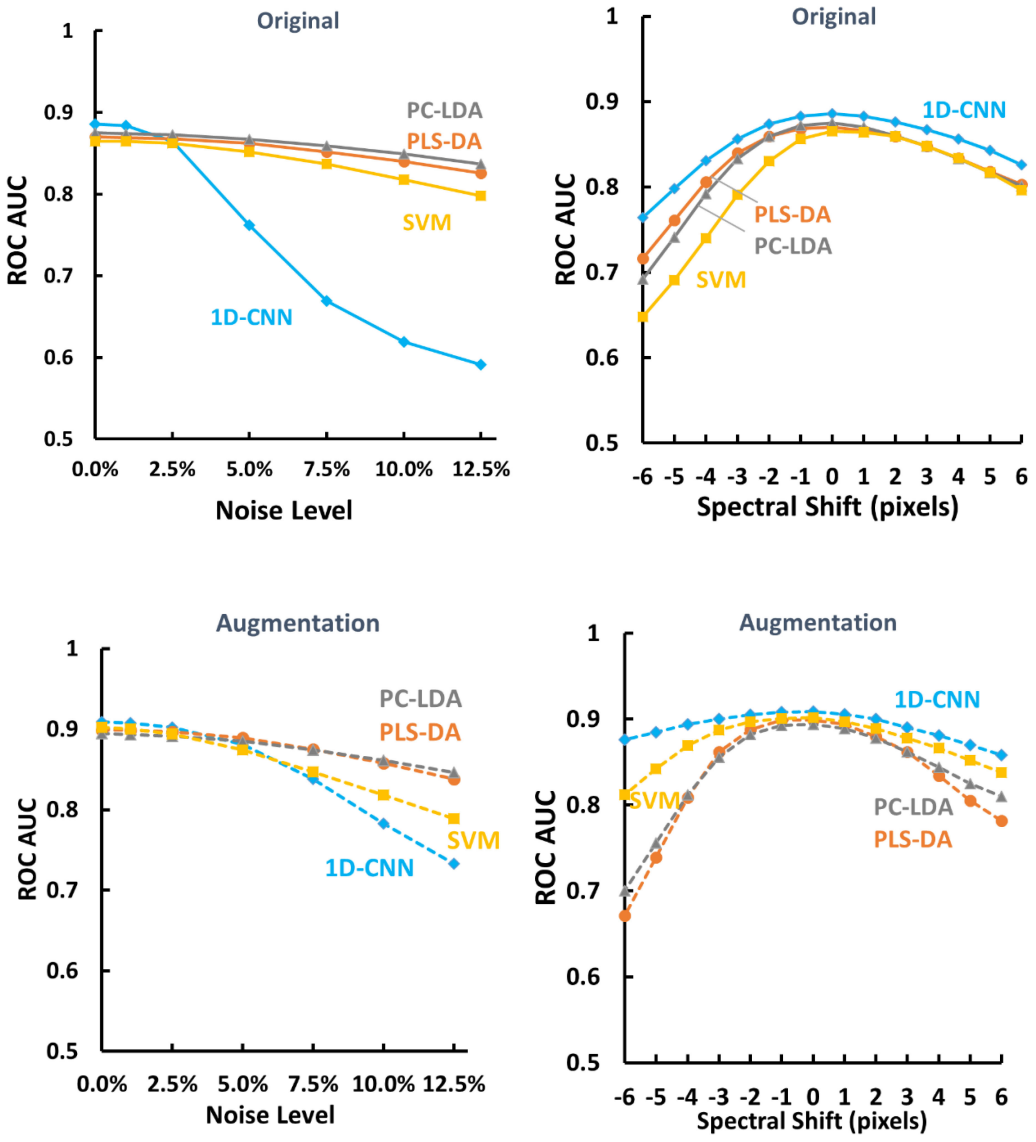
ROC AUC of the extended test dataset by adding random noise or spectral shift to the original test dataset for models based on the original training dataset without augmentation (top row) and models based on the original training dataset with augmentation (bottom row). Data shown are the mean of 56 random repetitions using parallel computing. 1D-CNN, one-dimensional convolutional neural networks; PLS-DA, partial least squares for discriminant analysis; PC-LDA, principal component and linear discriminant analysis; SVM, support vector machine.

When applying the models based on the original training dataset without augmentation (models in section 3.2) to the extended test dataset, the ROC AUCs of the extended test datasets are shown in **Figure 10** (top row). It is found that all the models including 1D-CNN, PLS-DA, PC-LDA and SVM perform the best on the original test dataset. They all can tolerate 2.5% of random noise or ±1 pixel shift; and the performance starts to deteriorate with further added noise or spectral shift. In terms of random noise, PC-LDA and PLS-DA perform better than 1D-CNN and SVM at high noise levels; while in terms of spectral shifting, 1D-CNN performs better than PC-LDA, PLS-DA and SVM across all the situations. It is also noticed that the effect for spectral shifting is not symmetric. The performance drops faster when the spectrum is shifted to lower wavenumbers.

#### 3.5.2 Models based on the original training dataset with augmentation

When applying the models based on the original training dataset with augmentation (models in section 3.3) to the extended test dataset, the ROC AUCs of the extended test datasets are shown in **Figure 10** (bottom row). Again, it is found that all the models including 1D-CNN, PLS-DA, PC-LDA and SVM perform the best on the original test dataset. The performance for models trained on the augmented training dataset was much better than the models trained on the original training dataset (**Supplementary Figures S1, S2**). In terms of random noise, PC-LDA and PLS-DA still perform better than 1D-CNN and SVM at high noise levels; while in terms of spectral shifting, 1D-CNN and SVM perform better than PC-LDA and PLS-DA. 1D-CNN still performs the best across all the situations.

## 4 Discussions

### 4.1 Data augmentation improved diagnostic performance of 1D-CNN and conventional machine learning techniques

Adding random noise, spectral shifting, linear combination of the spectra (65), and generative adversarial networks (67) were proposed in previous studies of deep neural networks, and none of them implemented all data augmentation strategies presented in this study. We applied all the data augmentation strategies to both deep neural networks and conventional machine learning techniques, and found that data augmentation not only improved the performance of deep neural networks, but also the conventional machine learning techniques. Particularly, data augmentation improved the performance of LR when the number of cases was less than the number of variables.

None of the previous studies provided details of how the data augmentation was implemented or evaluated the effect of different data augmentation strategies. In this study, we systematically investigated the effect of different data augmentation strategies and found that different augmentation strategies have variable contribution to the improvement of deep neural networks and conventional machine learning techniques. For example, augmentation by adding random noise improved SVM and LR, but not PLS-DA, PC-LDA and 1D-CNN; augmentation by spectral shifting improved LR but not PLS-DA, PC-LDA, SVM and1D-CNN; augmentation by spectral linear combination has almost no contribution to all the techniques (**Figure 7**); and augmentation by 1D-GAN improved the performance of 1D-CNN, PLS-DA, PC-LDA and SVM more than LR (**Table 2**). The best performance was achieved when all these strategies were combined (**Figure 9**).When all the augmentation strategies were combined, it improved the performance of 1D-CNN, PLS-DA, PC-LDA and SVM by 2–4%, and LR by 71%.

### 4.2 1D-CNN outperforms conventional machine learning approaches

Convolutional neural networks contain multiple hierarchical layers with each layer representing a specific data manipulation. It contains many parameters to train and thus very versatile. The versatility of convolutional neural networks brings both advantage and disadvantage, in that an optimal model can always be found by tuning the parameters, while in the meantime there is no standard architecture that can fit all situations. For example, in this study we tried 1D-CNN architectures of multiple convolutional layers with different number of kernels, kernel sizes, pooling methods, mini-batch sizes and sizes of the fully connected layer (section 2.3, **Supplementary Tables S1-S4**), and eventually found that 1D-CNN with 4 convolutional layers, with each convolutional layer having 16, 32, 64 and 128 kernels, kernel size = [3,1], mini-batch size = 256, and average pooling had the best performance (**Figure 4**). With the optimal 1D-CNN architecture and parameters, we found that 1D-CNN out-performed other machine learning techniques by 1–3% based on the original Raman spectra. After data augmentation, 1D-CNN outperformed other machine learning techniques by 1–2% (**Figure 9**).

Different from deep neural networks, conventional machine learning techniques were not as versatile as 1D-CNN, and thus there were less parameters to train. Therefore, in designing CNN, the performance of conventional machine learning techniques could be used as baseline for benchmarking convolutional neural networks. 1D-CNN outperforms conventional machine learning techniques, with the cost of longer training and more effort to find the optimal network architectures and parameters.

### 4.3 Parallel computing provides advantages for both 1D-CNN and conventional machine learning techniques

Cross validation is commonly used in conventional machine learning techniques (PLS-DA, PC-LDA, SVM and LR). The most commonly used cross validation techniques are leave-one-out cross-validation (LOO-CV) and K-fold cross-validation. For LOO-CV, usually a case or patient is left out for testing and the remaining n-1 cases or patients are used for training. The procedure is repeated n times so that every case or patient is tested once. The results are the average of the n models. Similar to LOO-CV, K-fold cross-validation is to divide the stratified cases or patients into K groups; one group of the cases or patients is left out for testing and the remaining K-1 groups are used for training. The procedure is repeated K times so that every case or patient is tested once. The results are the average of the K models. LOO-CV can be regarded as a special case of K-fold cross-validation where K=n.

Deep neural networks often require the dataset being split into training, validation and test datasets. The model is generated from the training dataset; the hyperparameters are fine-tuned from the validation dataset; and the test set provides unbiased evaluation of the final model. Sometimes the validation dataset can serve as the test dataset when the original dataset is divided into two subsets. If early stopping is needed for model training, an independent validation dataset is usually needed. LOO-CV and K-fold cross-validation have been used in deep neural networks for Raman spectrum classification (52, 54, 65). Some authors implemented K-fold cross-validation on the training dataset and tested on a holdout test set. When the test set is large enough and representative to the distribution of the dataset, it provides an unbiased evaluation of the model (31, 59, 73). However, as Khristoforova et al (74) pointed out, the major drawbacks of previous publications on Raman spectroscopy and chemometrics were insufficient sample size, lack of cross-validation, and/or incorrect division of the data into subsets. In this study, the original dataset was randomly split into training, validation and test datasets, and early stopping criteria were used during the model training process. This process was repeated 56 times, taking the advantage of parallel computing, and thus prevented over-fitting and bias.

### 4.4 Data augmentation improves model performance on extended test dataset

Although there are standard protocols for measurement and data processing of Raman spectra, including wavelength calibration, intensity calibration, fluorescence background removal, and/or normalization, it is still difficult to evaluate the performance of models across multiple systems (72, 75, 76). In this paper we proposed a simple method to evaluate the models on different conditions by adding random noise or spectral shift to the original test dataset to generate the extended test dataset, mimicking the situation of multiple systems. We evaluated the models trained on the original training dataset (section 3.2) and models trained on the augmented training dataset (section 3.3) to the original test dataset (**Figure 9**) and the extended test dataset (**Figure 10**). It was found that the models based on the original training dataset without augmentation performed well only on situations with similar spectral quality (i.e. original test dataset), and the performance became deteriorated when the spectral quality was compromised (i.e. the extended test dataset with increased random noise or spectral shifting) (**Figure 10**). However, the models based on the augmented training dataset not only improved the performance on the original test dataset (**Figures 9**, **10**), but also had higher tolerance on low spectral quality, i.e. the situations with increased random noise or spectral shifting (**Figure 10**). The models based on the augmented training dataset could perform even better on the extended test dataset (with up to 5% increase of random noise or 3-pixel spectral shifting) than the models based on the original training dataset applied to the original test dataset (**Supplementary Figures S1, S2**), indicating that data augmentation could improve the applicability of the models trained on high quality data to situations of low spectral quality.

## 5 Conclusion

In summary, we designed a one-dimensional convolutional neural networks (1D-CNN) for skin cancer detection by Raman spectroscopy and compared with conventional machine learning techniques (PLS-DA, PC-LDA, SVM and LR). We proposed and evaluated different data augmentation strategies including adding random noise, spectral shifting, spectral linear combination and synthetic spectra by 1D-GAN. Each augmentation strategy had different performance, but when all the augmentation strategies were combined, it substantially improved the performance of 1D-CNN and all the conventional machine learning analyses by 2–4% (p<0.0001, Wilcoxon). We also found that a well-designed 1D-CNN outperformed conventional machine learning techniques by 1–3% using original dataset and by 1–2% after data augmentation. Data augmentation is a simple but an effective way to improve the performance of deep neural networks and machine learning techniques. Models trained on augmented training dataset not only perform better, but also have higher tolerance on spectral quality of the test dataset.

## Data availability statement

Data can be made available to researchers upon application and subject to the approval of the Clinical Research Ethics Board of the University of British Columbia. Enquiries regarding data access can be directed to the corresponding author.

## Ethics statement

The studies involving humans were approved by the Clinical Research Ethics Board of the University of British Columbia. The studies were conducted in accordance with the local legislation and institutional requirements. The participants provided their written informed consent to participate in this study.

## Author contributions

JZ: Conceptualization, Data curation, Formal analysis, Investigation, Methodology, Software, Visualization, Writing – original draft, Writing – review & editing. HL: Conceptualization, Formal analysis, Funding acquisition, Investigation, Methodology, Project administration, Resources, Supervision, Writing – review & editing. SK: Investigation, Writing – review & editing. TL: Methodology, Writing – review & editing. HZ: Conceptualization, Formal analysis, Funding acquisition, Investigation, Methodology, Project administration, Resources, Supervision, Writing – review & editing.
