# Supplementary material for: Improving skin cancer detection by Raman spectroscopy using convolutional neural networks and data augmentation
Source: Front Oncol. 2024 Jun 19;14:1320220. doi: 10.3389/fonc.2024.1320220 (PMC11219827; doi:10.3389/fonc.2024.1320220)
Supplement: Supplementary file 1 [file DataSheet_1.pdf]

Improved skin cancer discrimination by Raman spectroscopy using convolutional neural networks and data augmentation

(Supplementary materials)

Jianhua Zhao[1,2], Harvey Lui[1,2], Sunil Kalia[1,3,4], Tim K. Lee[1,2] and Haishan Zeng[1,2] *

[1]Photomedicine Institute, Department of Dermatology and Skin Science, University of British Columbia and Vancouver Coastal Health Research Institute, Vancouver, Canada
[2]BC Cancer Research Institute, University of British Columbia, Vancouver, Canada
[3]BC Children's Hospital Research Institute, Vancouver, Canada
[4]Centre for Clinical Epidemiology and Evaluation, Vancouver Coastal Health Research Institute, Vancouver, Canada

Corresponding Author

Professor Haishan Zeng

BC Cancer Research Institute

University of British Columbia

675 West 10th Ave

Vancouver, BC V5Z 1L3

Email: hzeng@bccrc.ca

Phone: 604-675-8083

Supplementary Table S1.

Models of different number of convolutional layers, number of kernels of each convolutional layer and mini-batch sizes. ROC AUCs of the test dataset (mean ± standard deviation) based on models of different number of convolutional layers, number of kernels of each convolutional layer and mini-batch sizes. Original training dataset without augmentation, kernel size = [3,1], average pooling,

**Number of Convolutional Layers**	**Number of kernels of each convolutional layer**	**Mini-batch size**				
		**16**	**32**	**64**	**128**	**256**
1	16	0.866±0.028	0.861±0.030	0.863±0.028	0.865±0.028	0.871±0.026
	32	0.855±0.028	0.854±0.028	0.851±0.029	0.848±0.028	0.861±0.025
	64	0.855±0.031	0.847±0.027	0.845±0.028	0.844±0.028	0.862±0.026
	128	0.853±0.033	0.848±0.028	0.840±0.031	0.842±0.029	0.855±0.027
2	16,16	0.868±0.026	0.866±0.024	0.863±0.025	0.868±0.025	0.874±0.026
	32,32	0.865±0.026	0.862±0.027	0.862±0.026	0.863±0.025	0.869±0.025
	64,64	0.865±0.029	0.863±0.028	0.860±0.027	0.862±0.027	0.866±0.027
	128,128	0.859±0.032	0.856±0.033	0.861±0.027	0.859±0.027	0.858±0.030
	16,32	0.862±0.025	0.862±0.026	0.863±0.027	0.866±0.027	0.872±0.026
3	16,16,16	0.873±0.025	0.875±0.023	0.872±0.024	0.876±0.023	0.881±0.023
	32,32,32	0.870±0.028	0.862±0.027	0.861±0.028	0.862±0.029	0.870±0.027
	64,64,64	0.871±0.025	0.870±0.029	0.871±0.025	0.875±0.025	0.881±0.024
	128,128,128	0.873±0.024	0.875±0.027	0.868±0.028	0.875±0.025	0.875±0.027
	16,32,64	0.871±0.025	0.866±0.028	0.868±0.026	0.872±0.026	0.879±0.026
4	16,16,16,16	0.875±0.020	0.873±0.022	0.873±0.023	0.879±0.022	0.884±0.022
	32,32,32,32	0.872±0.025	0.873±0.023	0.871±0.024	0.875±0.024	0.877±0.024
	64,64,64,64	0.872±0.027	0.873±0.022	0.874±0.022	0.881±0.023	0.883±0.022
	128,128,128,128	0.875±0.025	0.873±0.025	0.871±0.026	0.878±0.023	0.882±0.024
	**16,32,64,128**	**0.876±0.024**	**0.876±0.022**	**0.877±0.024**	**0.884±0.023**	**0.886±0.022**
5	16,16,16,16,16	0.865±0.026	0.865±0.026	0.865±0.028	0.869±0.027	0.872±0.026
	32,32,32,32,32	0.873±0.027	0.871±0.028	0.871±0.025	0.871±0.026	0.874±0.025
	64,64,64,64,64	0.870±0.027	0.870±0.025	0.871±0.023	0.877±0.023	0.875±0.022
	128,128,128,128,128	0.872±0.025	0.872±0.021	0.872±0.022	0.876±0.024	0.871±0.025

pooling size = [2,1].

Supplementary Table S2.

Models of different kernel sizes. ROC AUCs of the test dataset (mean ± standard deviation) based on models of different number of convolutional layers, number of kernels of each convolutional layer and kernel sizes. Original training dataset without augmentation, mini-batch size = 256, average pooling, pooling size = [2,1].

Number of Convolutional Layers	Number of kernels of each convolutional layer	Kernel Size			
		[3,1]	[5,1]	[7,1]	[9,1]
1	16	0.871±0.026	0.864±0.025	0.857±0.025	0.862±0.026
2	16,32	0.872±0.026	0.870±0.025	0.873±0.026	0.870±0.028
3	16,32,64	0.879±0.026	0.875±0.024	0.877±0.024	0.874±0.026
4	16,32,64,128	0.886±0.022	0.874±0.024	0.879±0.022	0.881±0.023

Supplementary Table S3.

Models of different pooling methods. ROC AUCs of the test dataset (mean ± standard deviation) based on models of different number of convolutional layers, number of kernels of each convolutional layer and different pooling methods. Original training dataset without augmentation, pooling size = [2,1], mini-batch size = 256, kernel size = [3,1].

Number of Convolutional Layers	Number of kernels of each convolutional layer	Average-pooling	Max-pooling
1	16	0.871±0.026	0.870±0.026
2	16,32	0.872±0.026	0.874±0.026
3	16,32,64	0.879±0.026	0.876±0.025
4	16,32,64,128	0.886±0.022	0.876±0.023

Supplementary Table S4.

Models of different sizes of fully connected layer. ROC AUCs of the test dataset (mean ± standard deviation) based on models of different number of convolutional layers, number of kernels of each convolutional layer and sizes of fully connected layer. Original training dataset without augmentation, average pooling, pooling size = [2,1], mini-batch size = 256, kernel size = [3,1].

Number of Convolutional Layers	Number of kernels of each convolutional layer	Size of fully connected layer		
		128	256	512
1	16	0.869±0.026	0.871±0.026	0.870±0.025
2	16,32	0.873±0.025	0.872±0.026	0.871±0.027
3	16,32,64	0.880±0.026	0.879±0.026	0.878±0.026
4	16,32,64,128	0.884±0.024	0.886±0.022	0.880±0.024

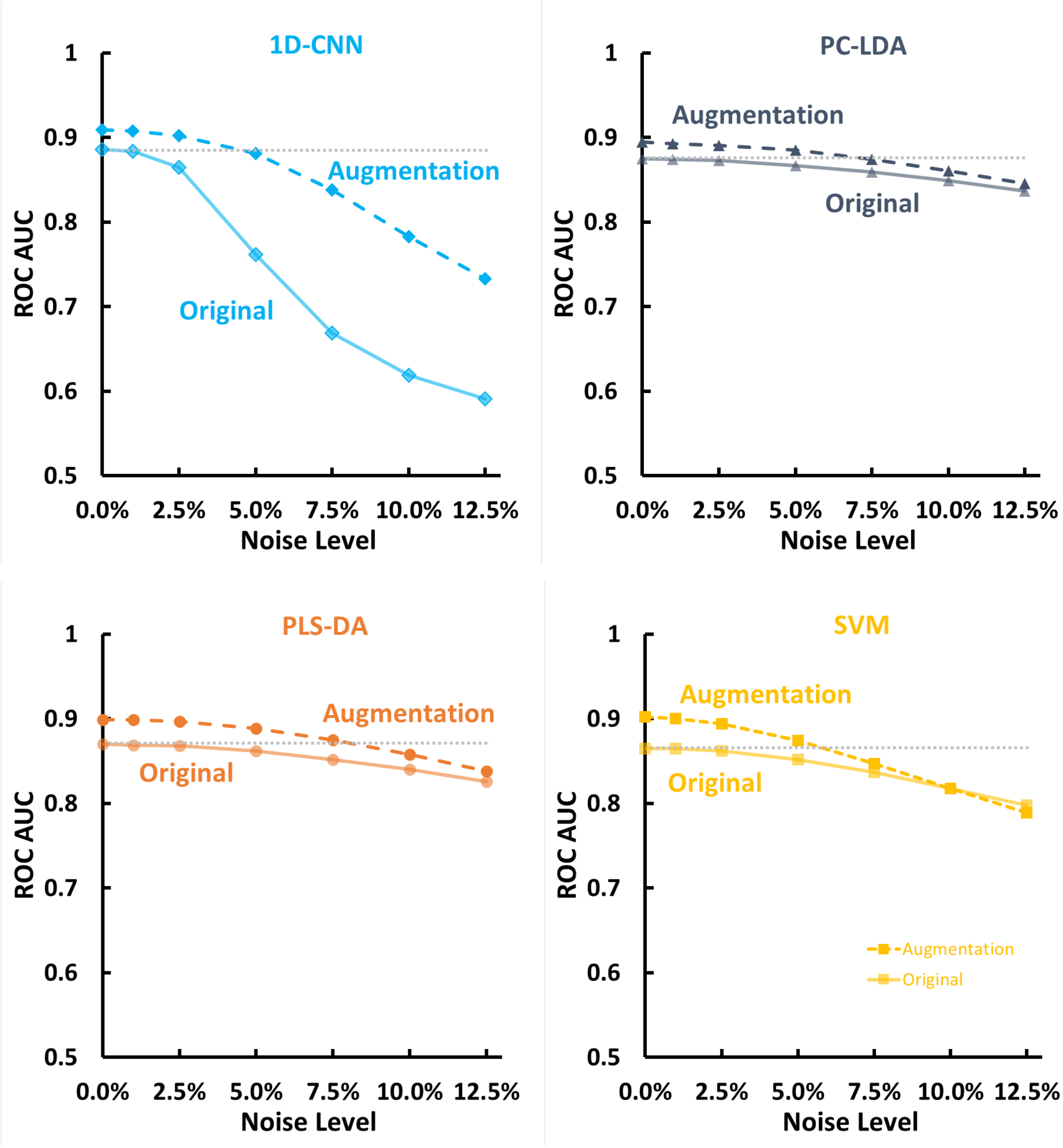

Supplementary Figure S1. Performance of the models based on the original training dataset (solid lines), and the original training dataset with augmentation (dashed lines), on the extended test dataset of different random noise levels. The dotted line indicates the performance of the models based on the original training dataset on the original test dataset. The improvement of data augmentation on all the models are apparent. 1D-CNN: one-dimensional convolutional neural networks; PC-LDA: principal component and linear discriminant analysis; PLS-DA: partial least squares for discriminant analysis; SVM: support vector machine.

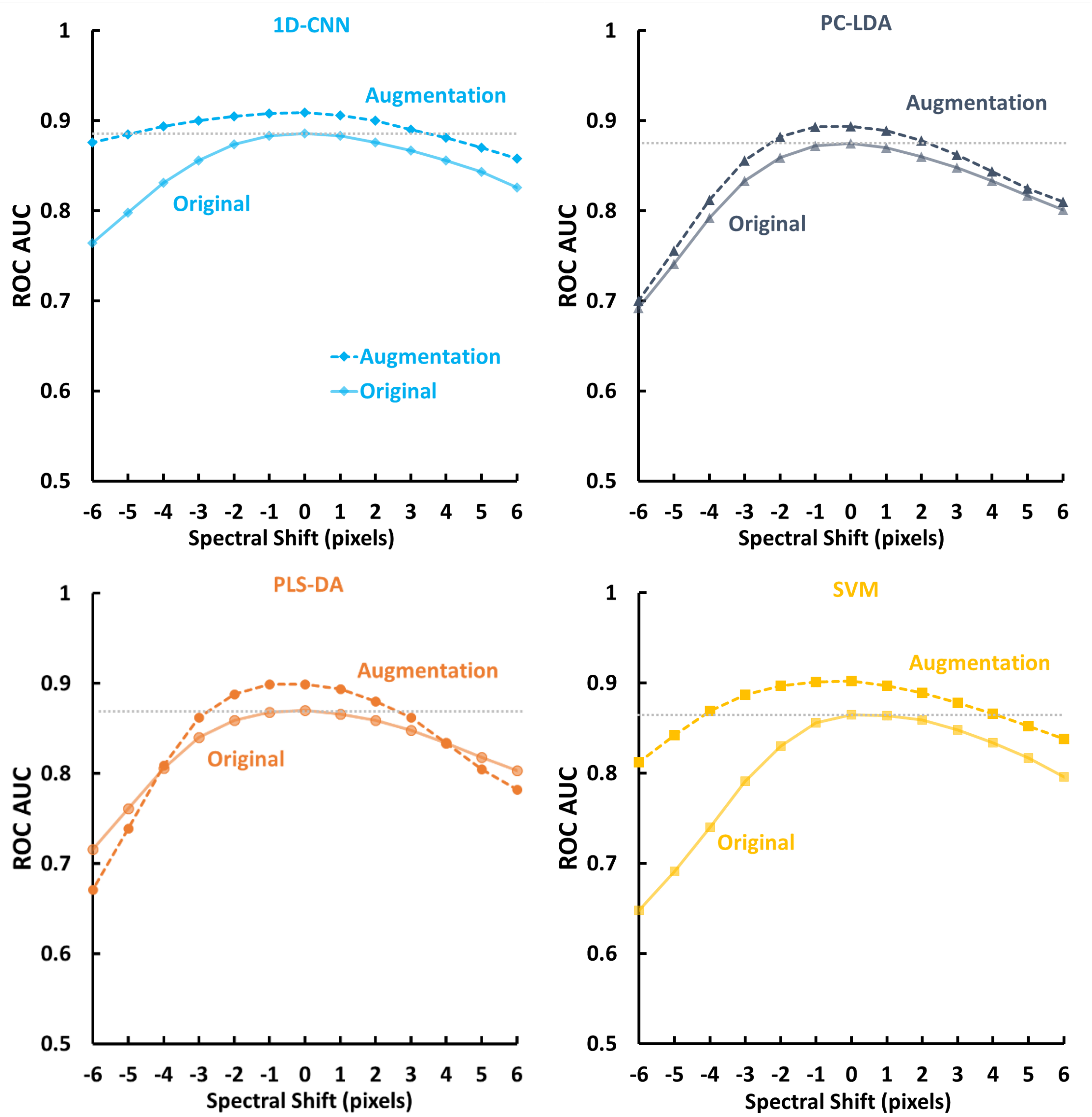

Supplementary Figure S2. Performance of the models based on the original training dataset (solid lines), and the original training dataset with augmentation (dashed lines), on the extended test dataset with spectral shifting. The dotted line indicates the performance of the models based on the original training dataset on the original test dataset. The improvement of data augmentation on all the models are apparent. 1D-CNN: one-dimensional convolutional neural networks; PC-LDA: principal component and linear discriminant analysis; PLS-DA: partial least squares for discriminant analysis; SVM: support vector machine.
